# Supplementary material for: Suppression of pathological oscillations with transcranial focused ultrasound in Parkinson’s disease
Source: Nat Commun. 2026 Mar 25;17:4471. doi: 10.1038/s41467-026-70714-7 (PMC13187342; doi:10.1038/s41467-026-70714-7)
Supplement: Supplementary file 1 — Supplementary Information [file 41467_2026_70714_MOESM1_ESM.pdf]

Appendix: Supplementary information

Table of Contents

Table S1: Acoustic and thermal properties used in the k-Wave simulations......2

Figure S1: Acoustic lens approach 3

Figure S2: Simulated and measured free field acoustic pressure maps.......4

Figure S3: Cortico-subthalamic Beta coherence 5

Figure S4: Temperature measurements during focused ultrasound in a porcine brain. 6

Table S2: Transducer positional error 8

Figure S6: Tremor amplitude for each participant 8

Figure S7: Post-hoc power calculations. 9

Figure S8 Mean change in reaction time (sensitivity analysis)......9

Material	Density (kg/m^3)	Sound speed (m/s)	Attenuation (db/cm)	Power coefficient	Thermal conductivity (W/(mK))	Specific heat capacity (J/(kgK))
Water/CSF[1]	1000	1488	0	1.05	0.6	4178
Soft tissue[1]	1045	1500	0.05	1.05	0.55	3696
Skull[1]	1732	2820	3.28	1.05	0.32	1313
Electrode[2]	21606	3417	0	1.05	31	134

Table S1: Acoustic and thermal properties used in the k-Wave simulations

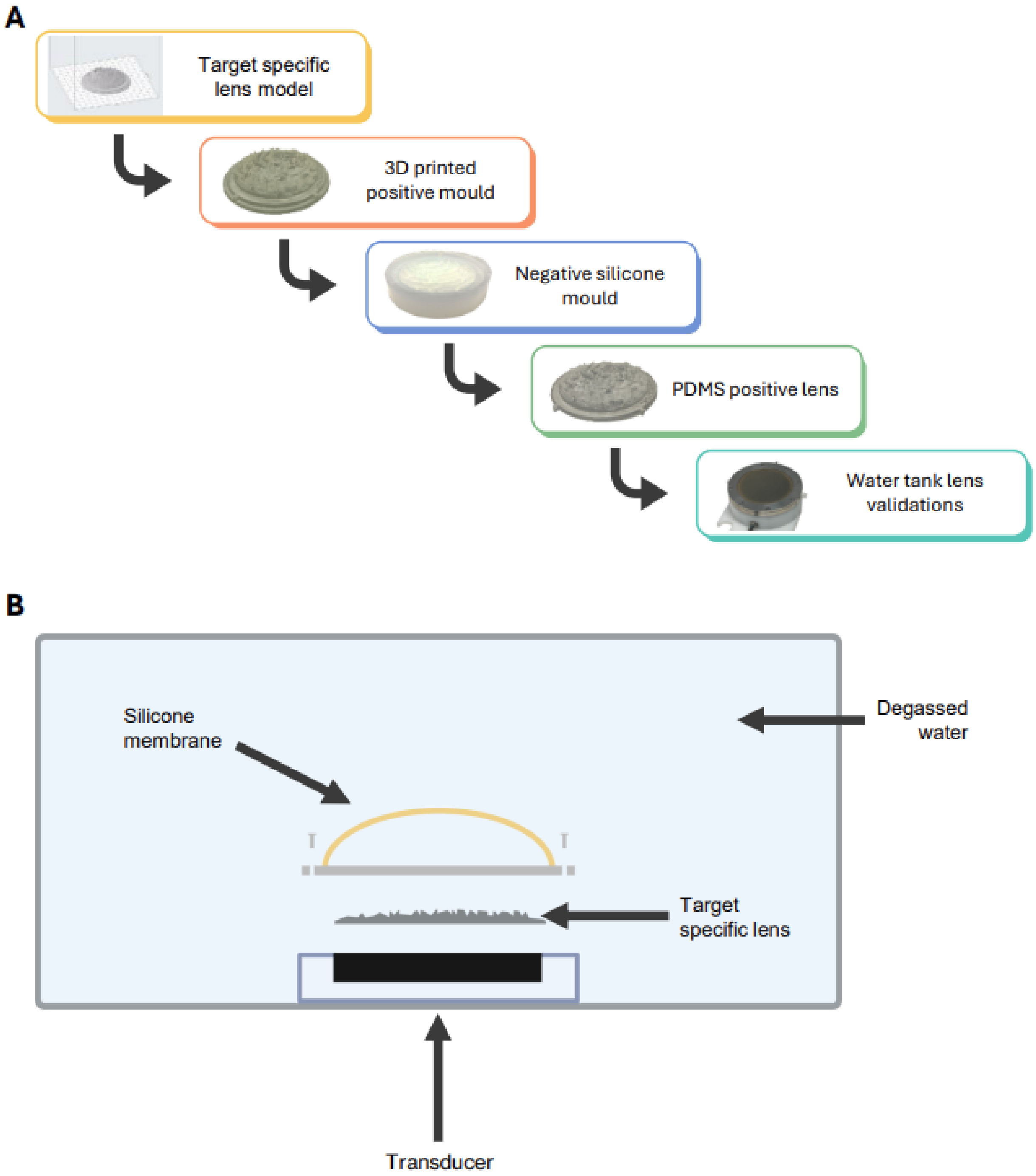

Figure S1: Acoustic lens approach. A) Overview of the target-specific acoustic lens manufacturing process. The process consists of the creation of a 3D lens model, 3D printing of a positive mould, creation of a negative silicone mould, the casting of the PDMS positive lens. **B) Lens mounting processes**. The target-specific lens and silicone membrane is mounted and secured in place under degassed water. Created in BioRender. Toth, J. (2026) https://www.biorender.com/zioat9b

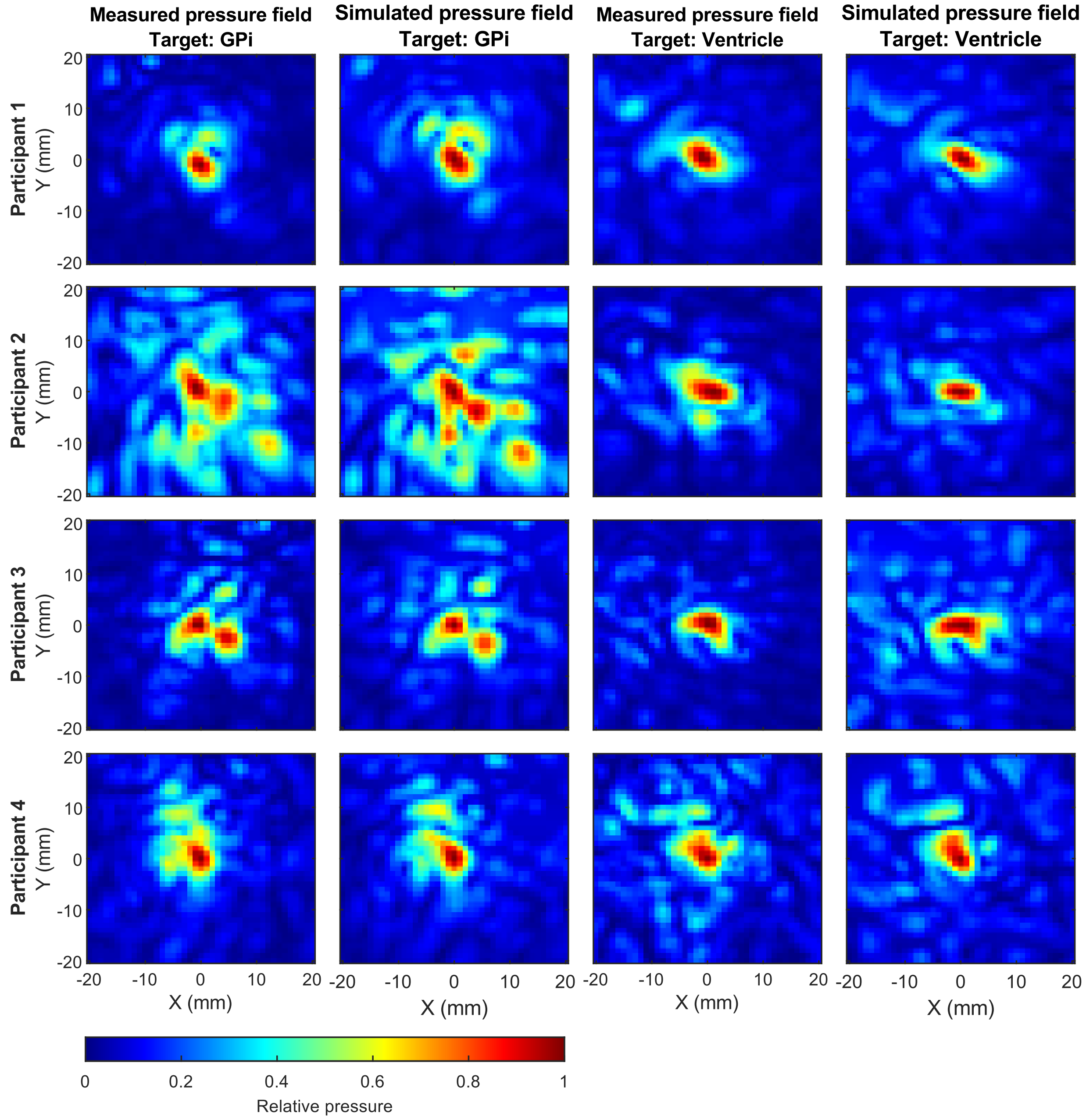

Figure S2: Simulated and measured free field acoustic pressure maps. Simulated and measured free field acoustic pressure maps across all four participants and the globus pallidus internus (GPi) and ventricle targets. Simulations were performed using the k-Wave toolbox with a 500kHz fundamental frequency. Measured pressure fields taken in a degassed water tank, measurements were taken with an ONDA HNA needle hydrophone. Each pixel represents the peak rarefaction pressure averaged over 10 pulses (500kHz, 20 cycles per pulse, 10ms pulse period). All pressure maps are displayed in the XY-plane at the axial depth (Z-axis) of maximum pressure with a 40mm x 40mm field of view and 1mm^2 spatial resolution. Pressure fields are individually normalised to the peak pressure within each map.

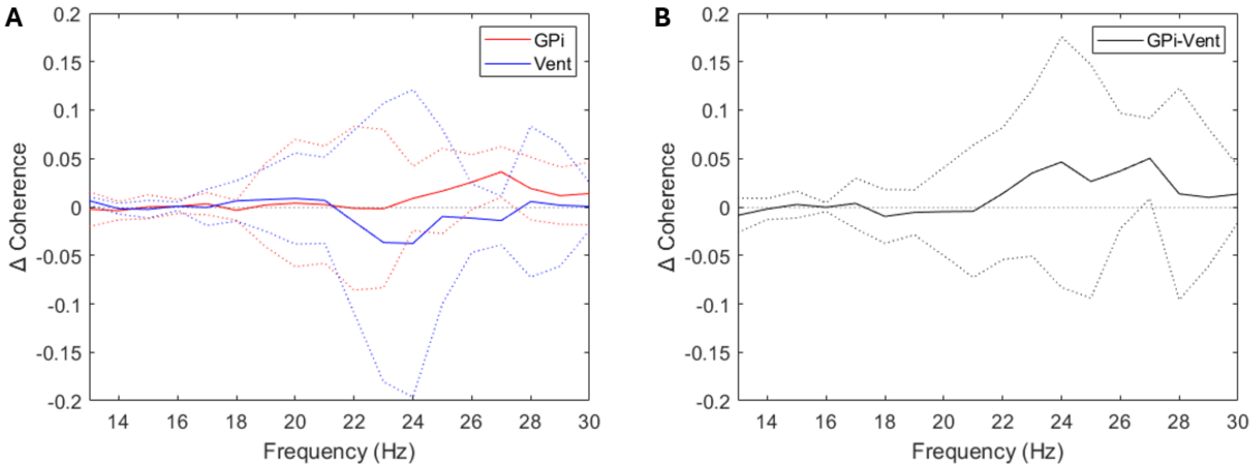

Figure S3: Cortico-subthalamic Beta coherence: A) Difference in M1-STN coherence: during TUS compared to baseline for GPi-TUS (red) and ventricle-TUS (blue). **B) Difference in change in M1-STN coherence:** change in coherence during ventricle-TUS subtracted from the change in coherence during GPi-TUS. The qualitative relative increase in high-beta coherence does not pass statistical significance. *Dotted lines = 95% confidence interval.*

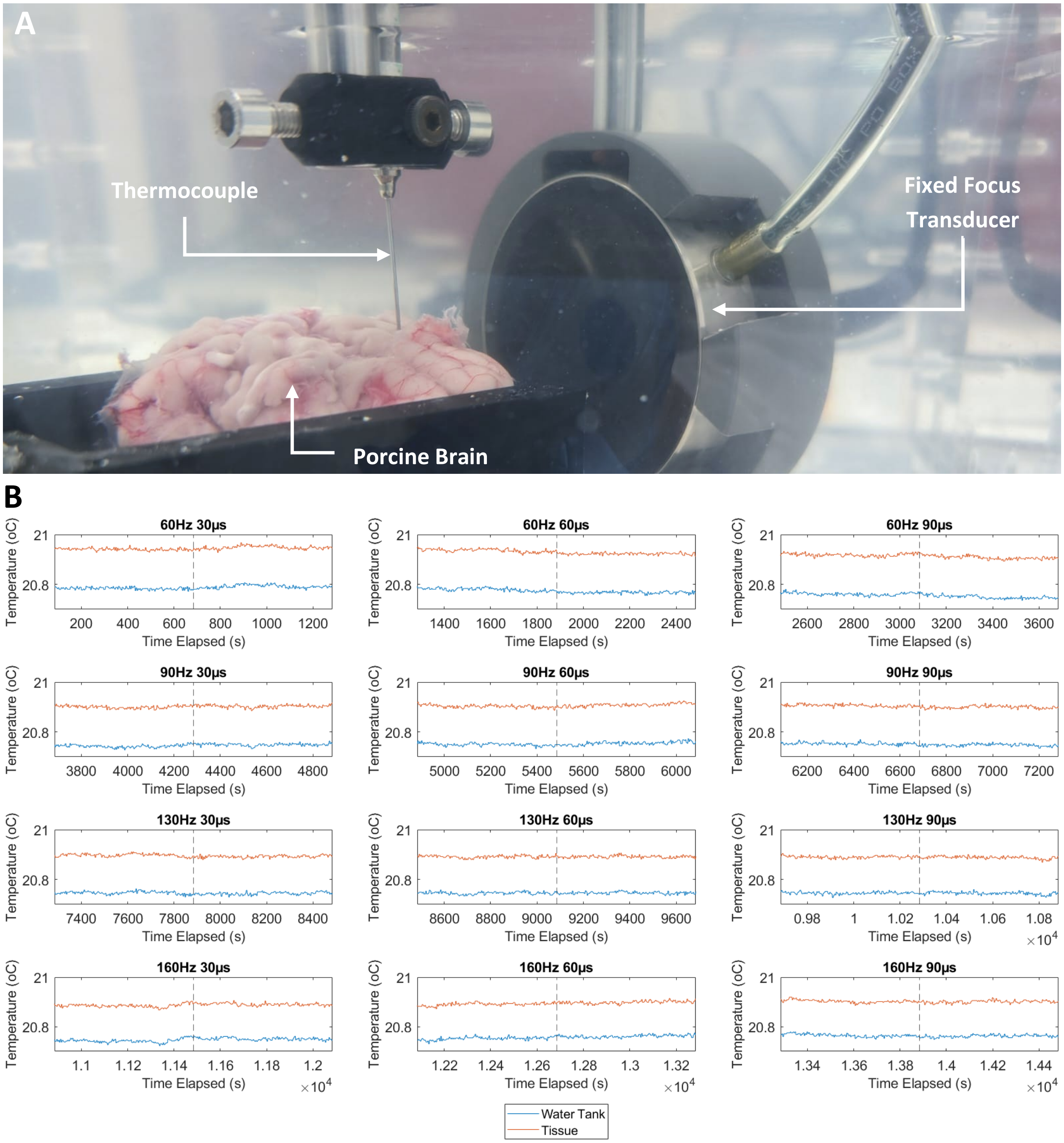

Figure S4: Temperature measurements during focused ultrasound in a porcine brain. A) Thermocouple tank set-up: Including the ultrasound transducer, thermocouple and porcine brain in a degassed water tank. A H107 single element transducer with a geometric focus sonic concepts transducer with a fundamental frequency of 500kHz was used as previously, with a focusing lens attached. 600kPa was produced at the thermocouple. **B) Temperature readings at a range of DBS inspired TUS parameters:** The tissue was sonicated with varying pulse lengths (30, 60 and 90µs) and pulse repetition frequencies (60, 90,

130 and 160Hz). The first 10 minutes is with TUS on and the second 10 minutes is with TUS off. Vertical dashed lines represents end of TUS on period for each condition, orange and blue lines represent temperature in brain tissue and water respectively.

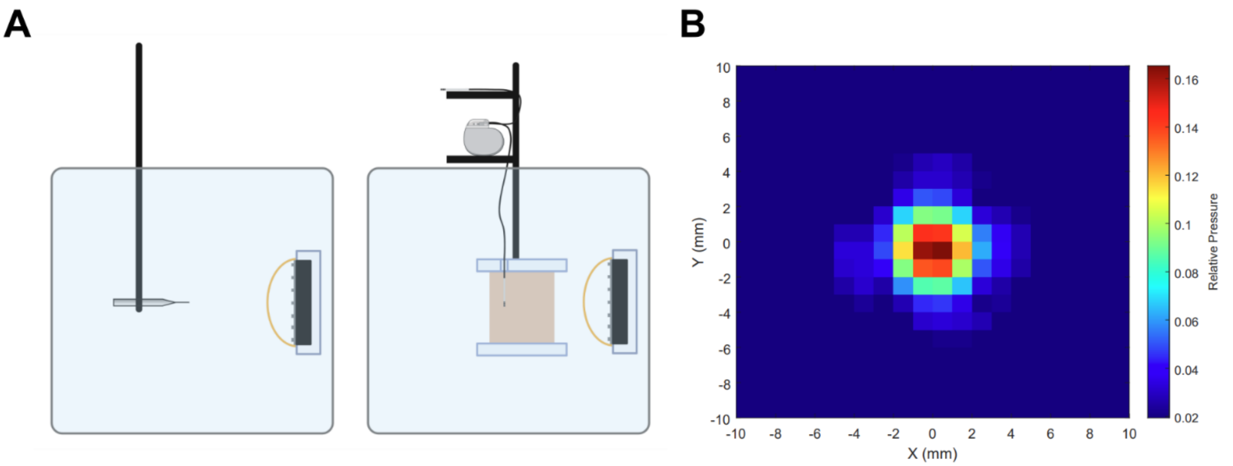

Figure S5: Ultrasound induced DBS artifact characterization: A) Pressure field measurement set-up: The TUS transducer immersed in degassed water with a focused lens affixed. The pressure field was measured with an ONDA HNA needle hydrophone. The left lead of a Medtronic Percept device with a 250Hz sampling rate was embedded in Agar, the right electrode was suspended in the air. During recording from the Medtronic Percept device the transducer was focused on contact 2, and recordings were taken from contact pair with the maximal artifact visible (0, 2). For each pressure and frequency condition recordings were taken for 5 minutes. *Created in BioRender. Toth, J. (2026) https://BioRender.com/rs2we2w* **B) The XY pressure map measured at the peak focus in the axial (Z-axis) plane with a 20mm x 20mm field of view and 1mm^2 resolution.** Each pixel represents the peak rarefaction pressure averaged over 10 pulses (500kHz, 40µs pulse duration, 10ms pulse period).

		Pariticpant 1		Pariticpant 2		Pariticpant 3		Pariticpant 4		Average	
		Ventricle	GPi	Ventricle	GPi	Ventricle	GPi	Ventricle	GPi	Ventricle	GPi
Target Error (mm)	Mean	0.41	0.41	0.59	0.63	0.57	0.48	0.46	0.49	0.51	0.50
	Std	0.32	0.33	0.48	0.38	0.45	0.31	0.23	0.31	0.37	0.33
Angular Error (degrees)	Mean	0.75	0.44	0.68	0.68	0.62	0.47	0.63	0.70	0.67	0.57
	Std	0.57	0.36	0.49	0.27	0.48	0.25	0.50	0.69	0.51	0.39
Twist Error (degrees)	Mean	-0.34	0.14	0.23	-0.02	0.35	-0.78	-0.65	-0.25	-0.11	-0.23
	Std	0.74	0.52	0.61	0.58	0.67	0.49	0.51	0.54	0.63	0.53

Table S2: Transducer positional error: Transducer positional error for each participant and each target during active stimulation in the rest block. Transducer position was tracked by a Brainsight TMS neuronavigation system (Rogue Research, Montréal, Canada). Positional error was calculated relative to the target trajectory, and comprises three components target error (translation), angular error (pitch) and twist error (roll). Error values were calculated at the beginning of each sonication pulse in participant 2-4, and continuously at 20Hz in participant 1 due to technical difficulties.

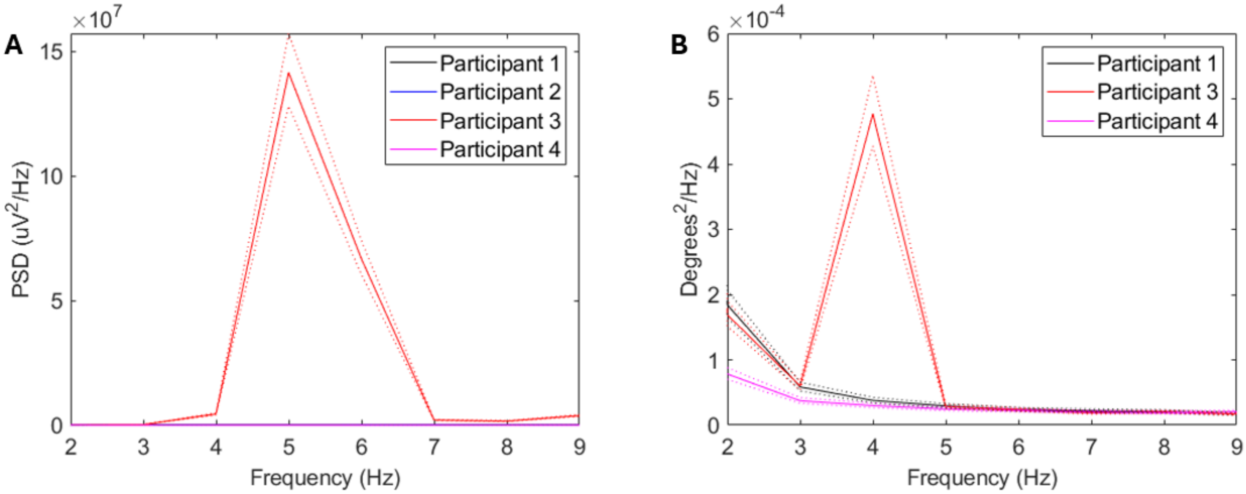

Figure S6: Tremor amplitude for each participant. Normalised power spectral density (PSD) for: A) Right arm accelerometry data. B) Continuous streaming head tracker data. Note that continuous streaming data was unavailable for participant 2. *Dotted line = 95% confidence interval*

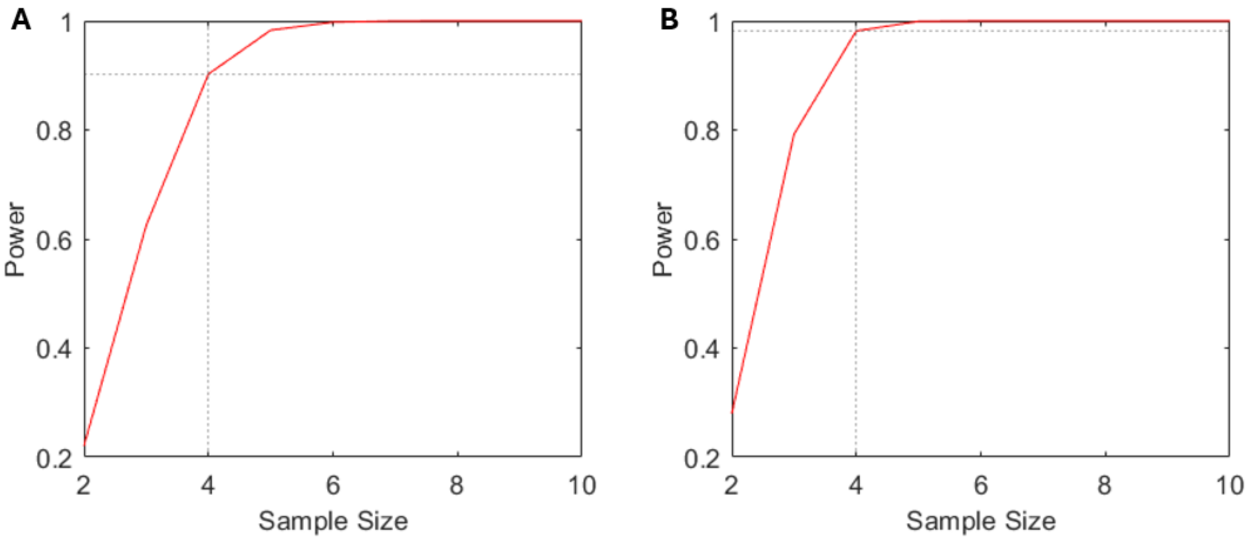

Figure S7: Post-hoc power calculations. A) Beta-power reduction in subthalamic nucleus. Power = 0.90 with a sample size of four. **B) Change in reaction time.** Power = 0.98 with a sample size of four.

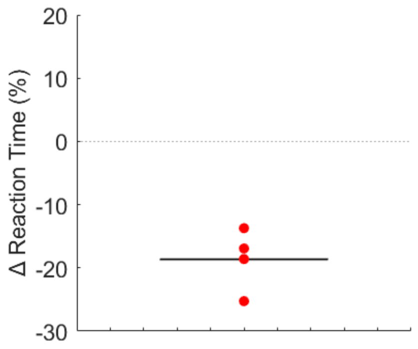

Figure S8: Mean change in reaction time (sensitivity analysis): Sensitivity analysis to assess for influence of outliers on change in reaction time (Mean = -18.63% (CI: -10.89 to -26.36, p = 0.0046). Note that main reaction time analysis presented in manuscript used the median reaction time for each participant.

References:

1. Butler, C.R., *et al*. Transcranial ultrasound stimulation to human middle temporal complex improves visual motion detection and modulates electrophysiological responses. *Brain Stimul* **15**, 1236-1245 (2022).
2. Elwassif, M.M., Kong, Q., Vazquez, M. & Bikson, M. Bio-heat transfer model of deep brain stimulation-induced temperature changes. *J Neural Eng* **3**, 306-315 (2006).
