# Supplementary material for: Suppression of pathological oscillations with transcranial focused ultrasound in Parkinson’s disease
Source: Nat Commun. 2026 Mar 25;17:4471. doi: 10.1038/s41467-026-70714-7 (PMC13187342; doi:10.1038/s41467-026-70714-7)
Supplement: Supplementary file 2 — Reporting Summary [file 41467_2026_70714_MOESM2_ESM.pdf]

nature portfolio

Corresponding author(s): John Eraifej: john.eraifej@univ.ox.ac.uk

Last updated by author(s): Jan 16, 2026

Reporting Summary

Nature Portfolio wishes to improve the reproducibility of the work that we publish. This form provides structure for consistency and transparency in reporting. For further information on Nature Portfolio policies, see our Editorial Policies and the Editorial Policy Checklist.

Statistics

For all statistical analyses, confirm that the following items are present in the figure legend, table legend, main text, or Methods section.

n/a	Confirmed	
☐	☒	The exact sample size (*n*) for each experimental group/condition, given as a discrete number and unit of measurement
☐	☒	A statement on whether measurements were taken from distinct samples or whether the same sample was measured repeatedly
☐	☒	The statistical test(s) used AND whether they are one- or two-sided<br>*Only common tests should be described solely by name; describe more complex techniques in the Methods section.*
☐	☒	A description of all covariates tested
☐	☒	A description of any assumptions or corrections, such as tests of normality and adjustment for multiple comparisons
☐	☒	A full description of the statistical parameters including central tendency (e.g. means) or other basic estimates (e.g. regression coefficient) AND variation (e.g. standard deviation) or associated estimates of uncertainty (e.g. confidence intervals)
☐	☒	For null hypothesis testing, the test statistic (e.g. *F*, *t*, *r*) with confidence intervals, effect sizes, degrees of freedom and *P* value noted<br>*Give P values as exact values whenever suitable.*
☒	☐	For Bayesian analysis, information on the choice of priors and Markov chain Monte Carlo settings
☒	☐	For hierarchical and complex designs, identification of the appropriate level for tests and full reporting of outcomes
☒	☐	Estimates of effect sizes (e.g. Cohen's *d*, Pearson's *r*), indicating how they were calculated

Our web collection on statistics for biologists contains articles on many of the points above.

Software and code

Policy information about availability of computer code

Data collection	The ultrasound stimulation waveform was digitally generated using Python 3.8.10.<br>Auditory masks were generated using the simpleaudio package for Python 3.8.10.<br>Tasks displayed to the participants were generated with PsychoPy 2024.2.4 with Python 3.8.10.<br>Neuronavigation data was collected with Brainsight v2.5.5.<br>Local field potentials were recorded using Medtronic Percept's indefinite streaming mode.
Data analysis	Ultrasound simulations were performed using the k-Wave toolbox in MATLAB.<br>Local field potential (LFP) data were converted to MAT-files from JSON files using the Perceive Toolbox in MATLAB.<br>All data processing (including electroencephalography, LFP and behavioural data) and statistical analyses were performed using MATLAB.<br>PROBTRACX was used to perform probabilistic tractrography.<br>MRI preprocessing was performed using FSL, including the use of the FLIRT and FNIRT packages for registration.<br>BEDPOSTX was used for ball and stick modelling of local diffusion parameters.<br>Statistical tests were two-sided - we note that this is not explicit in the manuscript and will be added if a revision is offered

For manuscripts utilizing custom algorithms or software that are central to the research but not yet described in published literature, software must be made available to editors and reviewers. We strongly encourage code deposition in a community repository (e.g. GitHub). See the Nature Portfolio guidelines for submitting code & software for further information.

Data

Policy information about availability of data

All manuscripts must include a data availability statement. This statement should provide the following information, where applicable:

- Accession codes, unique identifiers, or web links for publicly available datasets
- A description of any restrictions on data availability
- For clinical datasets or third party data, please ensure that the statement adheres to our policy

This is an interim analysis of an ongoing study so data cannot be made available at this stage but can be shared on request once the study has been completed.

Research involving human participants, their data, or biological material

Policy information about studies with human participants or human data. See also policy information about sex, gender (identity/presentation), and sexual orientation and race, ethnicity and racism.

Reporting on sex and gender	Sex was reported, all four participants were male.
Reporting on race, ethnicity, or other socially relevant groupings	N/A
Population characteristics	Ages: 54, 63, 55 and 54.
Recruitment	Four consecutive patients were recruited from DBS follow-up clinics (Table 1). Patients were eligible for participation if they had PD, DBS targeted to the STN, had a Percept® implanted pulse generator (IPG), and had a measurable beta peak on screening. Potential participants were excluded if they could not tolerate having their DBS turned off for the duration of the experiment, had an extreme language barrier and/or had a neurological/psychiatric comorbidity that may confound the results. Patients were recruited once they were on stable dopaminergic medications and had stable DBS settings.
Ethics oversight	This study was sponsored by the University of Oxford and conducted after NHS Research Ethics Committee approval (Reference: 23/EM/0165) and registration (trial registration no. NCT06932185), in accordance with the Declaration of Helsinki.

Note that full information on the approval of the study protocol must also be provided in the manuscript.

Field-specific reporting

Please select the one below that is the best fit for your research. If you are not sure, read the appropriate sections before making your selection.

☐ Life sciences ☒ Behavioural & social sciences ☐ Ecological, evolutionary & environmental sciences

For a reference copy of the document with all sections, see nature.com/documents/nr-reporting-summary-flat.pdf

Behavioural & social sciences study design

All studies must disclose on these points even when the disclosure is negative.

Study description	Quantitative experimental
Research sample	Parkinson's patients who had DBS capable of recording implanted.
Sampling strategy	No sample size calculation was performed.
Data collection	Computerized task performance was collected using a mouse, Unified Parkinson's Disease Rating Scale part III (UPDRS-III) assessments were collected by a blinded assessor.
Timing	UPDRS-III assessment was completed with each participant at baseline each morning and at the end of each sham and active block. Computerized tasks were performed using sham and active tasks.
Data exclusions	N/A
Non-participation	N/A
Randomization	In this randomised controlled cross-over study the order of sessions (active or sham) were randomised.

Reporting for specific materials, systems and methods

We require information from authors about some types of materials, experimental systems and methods used in many studies. Here, indicate whether each material, system or method listed is relevant to your study. If you are not sure if a list item applies to your research, read the appropriate section before selecting a response.

Materials & experimental systems

n/a	Involved in the study
☒	☐ Antibodies
☒	☐ Eukaryotic cell lines
☒	☐ Palaeontology and archaeology
☒	☐ Animals and other organisms
☒	☐ Clinical data
☒	☐ Dual use research of concern
☒	☐ Plants

Methods

n/a	Involved in the study
☒	☐ ChIP-seq
☒	☐ Flow cytometry
☐	☒ MRI-based neuroimaging

Plants

Seed stocks

Report on the source of all seed stocks or other plant material used. If applicable, state the seed stock centre and catalogue number. If plant specimens were collected from the field, describe the collection location, date and sampling procedures.

Novel plant genotypes

Describe the methods by which all novel plant genotypes were produced. This includes those generated by transgenic approaches, gene editing, chemical/radiation-based mutagenesis and hybridization. For transgenic lines, describe the transformation method, the number of independent lines analyzed and the generation upon which experiments were performed. For gene-edited lines, describe the editor used, the endogenous sequence targeted for editing, the targeting guide RNA sequence (if applicable) and how the editor was applied.

Authentication

Describe any authentication procedures for each seed stock used or novel genotype generated. Describe any experiments used to assess the effect of a mutation and, where applicable, how potential secondary effects (e.g. second site T-DNA insertions, mosiacism, off-target gene editing) were examined.

Magnetic resonance imaging

Experimental design

Design type

Structural images.

Design specifications

N/A

Behavioral performance measures

N/A

Acquisition

Imaging type(s)

T1-weighted MPRAGE and diffusion weighted

Field strength

3T

Sequence & imaging parameters

Structural T1-weighted MPRAGE sequences were acquired (repetition time (TR) = 2659ms, echo time (TE) = 2.93ms, inversion time = 1058ms, flip angle (FA) = 8°, field of view = 256 x 256mm, voxel size = 1 x 1 x 1mm3).

Multiband diffusion weighted sequence achieved a voxel size of 1.8 x 1.8 x 1.8mm3. Diffusion weighting was applied along 109 non-colinear gradient directions (b = 500s/mm2 for 15 volumes, 1000s/mm2 for 30 volumes, 2600 s/mm2 for 64 volumes), with five non-diffusion (b = 0) weighted volumes (TR = 6233ms, TE = 73.9ms, FA = 90°).

Area of acquisition

Brain

Diffusion MRI ☒ Used ☐ Not used

Parameters

Multiband diffusion weighted sequence achieved a voxel size of 1.8 x 1.8 x 1.8mm3. Diffusion weighting was applied along 109 non-colinear gradient directions (b = 500s/mm2 for 15 volumes, 1000s/mm2 for 30 volumes, 2600 s/mm2 for 64 volumes), with five non-diffusion (b = 0) weighted volumes (TR = 6233ms, TE = 73.9ms, FA = 90°). An additional b = 0 image was acquired with an opposing phase encoding direction was also acquired for distortion correction.

Preprocessing

Preprocessing software

Image pre-processing was carried out using the FMRIB Software Library (FSL; Oxford, UK). BEDPOSTX was used for ball and stick modelling of local diffusion parameters, with up to three crossing fibres per voxel.

Normalization

To generate native space binarised masks pre-operative T1-weighted MRI images were registered to post-operative CT images using FLIRT and registered to Montreal Neurological Institute (MNI) standard space using FNIRT. The inverse warp was then applied to the left STN and left GPi masks in the DISTAL atlas, and these were then binarised to produce left STN and left

	GPi masks in native space.
Normalization template	MNI standard space and DISTAL atlas.
Noise and artifact removal	In the T1-weighted scans susceptibility-induced off-resonance field was estimated using topup using b = 0 volumes with opposing PE directions. Eddy was then used to correct for motion and eddy currents. In the diffusion weighted scans an additional b = 0 image was acquired with an opposing phase encoding direction was also acquired for distortion correction.
Volume censoring	N/A

Statistical modeling & inference

Model type and settings	N/A
Effect(s) tested	N/A

Specify type of analysis: ☐ Whole brain ☐ ROI-based ☐ Both

Statistic type for inference (See Eklund et al. 2016)	N/A
Correction	N/A

Models & analysis

n/a	Involved in the study
☒	☐ Functional and/or effective connectivity
☒	☐ Graph analysis
☒	☐ Multivariate modeling or predictive analysis
